# Preparation of Oral Artesunate-Chitosan Oligosaccharide–Retinoic Acid Copolymer Micelles for Attenuating Hepatic Fibrosis

**DOI:** 10.3390/pharmaceutics18060682

**Published:** 2026-05-29

**Authors:** Shiyuan Lin, Feixian Lu, Qiao Li, Kefeng Zhang, Wei Zhang, Hui Chen, Jianxin Wang

**Affiliations:** 1 Science and Technology Innovation Center, Guangzhou University of Chinese Medicine, Guangzhou 510405, China; sylin@glmu.edu.cn; 2 College of Pharmacy, Guilin Medical University, Guilin 541199, China; 3 Key Laboratory of Pharmacology for Prevention and Treatment of High Incidence Diseases in Guangxi Higher Education Institutions, Guilin Medical University, Guilin 541199, China; 4 Department of Pharmaceutics, School of Pharmacy, Fudan University and Key Laboratory of Smart Drug Delivery, Ministry of Education, Shanghai 201203, China

**Keywords:** chitosan oligosaccharide, artesunate, self-assembled micelles, hepatic fibrosis, ferroptosis, oral absorption

## Abstract

**Background**: Hepatic fibrosis is characterized by the abnormal activation of hepatic stellate cells (HSCs) and excessive deposition of the extracellular matrix. Currently, effective clinical therapeutic strategies remain limited. Modulating ferroptosis-related pathways in activated HSCs has emerged as a promising therapeutic target for hepatic fibrosis treatment. **Methods**: An amphiphilic copolymer was synthesized by conjugating COS with ART, which spontaneously self-assembled into micelles; subsequent modification with retinoic acid (RA) yielded RA-functionalized ART–COS copolymer micelles. Curcumin was selected as a model drug to evaluate the potential of the micelles in enhancing intestinal epithelial transport, oral absorption and bioavailability. Meanwhile, in vitro targeting ability, capacity to modulate ferroptosis in HSCs and in vivo therapeutic efficacy were systematically investigated. **Results**: The RA-functionalized ART–COS micelles significantly enhanced intestinal epithelial drug transport, oral absorption, and bioavailability. In vitro experiments demonstrated that the micelles preferentially accumulate in activated HSCs, inhibit GPX4 expression, and induce excessive ROS production and ferroptosis, thereby effectively attenuating hepatic fibrosis. In vivo studies confirmed that the micelles regulated extracellular matrix metabolism, reduced collagen deposition, suppressed the activation and proliferation of HSCs, and ultimately helped attenuate hepatic fibrosis progression. **Conclusions**: This study successfully developed RA-functionalized ART–COS copolymer micelles. The micelles improve the accumulation of artesunate in liver tissue and yield favorable anti-fibrotic effects, thereby providing a promising translational strategy for anti-fibrotic therapy.

## 1. Introduction

Hepatic fibrosis represents one of the most prominent manifestations of chronic liver disease, a progressive inflammatory disorder triggered by multiple etiological factors including alcohol abuse, hepatitis B virus infection, non-alcoholic steatohepatitis, and cholestasis [1]. A hallmark of the fibrotic process is the excessive accumulation of the extracellular matrix (ECM), which is accompanied by complex pathophysiological alterations. If left untreated, hepatic fibrosis can further progress to cirrhosis and even hepatocellular carcinoma, thereby severely endangering human health [2,3,4,5,6]. Notably, accumulating clinical evidence has confirmed that hepatic fibrosis is a reversible process [7,8,9], emphasizing the significance of early detection and appropriate intervention strategies for its effective treatment and reversal.

The molecular pathogenesis of hepatic fibrosis is not yet fully elucidated. Hepatic stellate cells (HSCs), located in the Disse space between hepatic sinusoidal endothelial cells and hepatocytes, are considered the main drivers of hepatic fibrosis and possess plasticity, which can regulate liver growth, immunity, and inflammation [10]. Their activation, proliferation, and transdifferentiation into myofibroblasts are the core and key links in the process of hepatic fibrosis [11,12,13]. Current anti-fibrotic research mainly targets HSCs, which can be activated by a variety of molecules and signaling pathways, such as TGF-β/Smad, PDGF/ERK, Growth factor/mTOR, NF-κB, and others [14,15,16,17,18]. Drugs can alleviate and inhibit the progression of hepatic fibrosis by blocking inflammatory signaling pathways. Hepatocytes are the main sites for iron storage in the body, and recent studies have found that the levels of iron ions and lipid peroxidation in the liver after chronic injury are significantly increased, indicating a close relationship between hepatocyte iron metabolism disorders and hepatic fibrosis [19,20,21]. Ferroptosis is a new mode of cell death regulation caused by iron overload, redox homeostasis disorder, and increased lipid peroxidation, mainly through the inhibition of glutathione peroxidase 4 or cystine/glutamate antiporter activity, decreasing the generation of intracellular reducing substances such as glutathione, thereby breaking the cellular redox balance, inducing lipid peroxidation of the cell membrane and membrane perforation, and leading to cancer cell death [22,23,24]. Inducing ferroptosis in HSCs may be a potential target for the treatment of hepatic fibrosis [25,26,27,28,29]. Curcumin is a natural polyphenolic compound extracted from the traditional Chinese medicine turmeric, with a wide range of pharmacological effects such as antioxidant, anti-inflammatory, hepatoprotective, free radical scavenging, and inhibition of tumor proliferation [30,31,32,33]. Curcumin can downregulate the level of glutathione peroxidase 4, leading to lipid peroxidation and reactive oxygen species accumulation, thereby modulating ferroptosis and exhibiting good anti-cancer properties [34,35,36].

Artesunate is a semi-synthetic derivative of the traditional Chinese medicine antimalarial artemisinin, containing a unique peroxide bridge structure, which is the esterification product of dihydroartemisinin and succinic anhydride. It possesses pharmacological effects such as antimalarial, anti-inflammatory, antitumor, anti-fibrotic, and immune function regulation. Recent research has found that artesunate can also be used to treat liver diseases [37,38,39,40]. On one hand, it activates lysosomes and increases the concentration of iron ions by enhancing ferritin hydrolysis, thereby inducing ferroptosis in an iron-dependent manner. This pathway can effectively inhibit the proliferation of HSCs, promote apoptosis of activated cells, and reduce collagen production [41,42]. At the same time, artesunate can significantly increase the content of ceramide in HSCs; enhance the expression of PPAR-γ, p53, and caspase3; inhibit cell proliferation; and promote cell apoptosis [43,44,45].

Chitosan oligosaccharides are small molecular water-soluble polysaccharides derived from the degradation of chitosan, with many active groups such as amino and hydroxyl groups. They offer advantages such as abundantly available sourcing, safety and non-toxicity, good biocompatibility, and degradability. They display anti-inflammatory and antibacterial effects and can be used as medical dressings to promote wound repair. More importantly, they can also serve as drug delivery carriers [46,47,48]. It has been reported that chitosan oligosaccharides also have strong immune-modulating and hepatoprotective effects. They can regulate macrophage polarization by inhibiting the JAK/STAT pathway, alleviate CCl_4_-induced hepatic fibrosis, and enhance the release of matrix metalloproteinase I from fibroblasts to promote ECM degradation [49]. Additionally, chitosan oligosaccharides form nanoparticles with positive charge, displaying the strongest liver enrichment ability and facilitating penetration in the liver area of the body [50,51]. Building upon this foundation, we proposed to utilize the reactive groups on the chitosan oligosaccharide molecule to covalently conjugate with the carboxyl groups of the artesunate molecule, synthesizing an amphiphilic polymer that can self-assemble into micelles. So far, there have been no reports on the drug delivery system of covalently coupled chitosan oligosaccharide–artesunate conjugates.

HSCs express retinoic acid receptors (RARs) and retinoic acid X receptors (RXRs), with all-trans retinoic acid showing high affinity for RARs [52]. Retinoic acid deficiency is closely related to the progression of hepatic fibrosis. As an endogenous ligand for retinoic acid receptors, studies have indicated that retinoic acid can also be used as a drug for the treatment of hepatic fibrosis [53,54,55].

In brief, we hypothesized that conjugating artesunate to chitosan oligosaccharide to form self-assembled nanomicelles, combined with surface modification with retinoic acid, can enhance the solubility, oral absorption, and HSCs uptake efficiency of the drug. In this study, curcumin, a drug with poor solubility and permeability, was selected as the model compound to investigate the intestinal absorption mechanisms of a novel nanomicelle carrier, its targeting ability for HSCs, and its underlying mechanism of reversing hepatic fibrosis. Our study is designed to address the treatment of chronic liver diseases through the development of novel strategies based on advanced drug delivery platforms.

## 2. Materials and Methods

### 2.1. Materials

Chitosan oligosaccharide (COS, product code: C3925; molecular weight: 3300 Da; deacetylation degree: 90.5%) was purchased from Hefei Bomei Biotechnology Co., Ltd. (Hefei, China). Artesunate (ART), all-trans retinoic acid (RA), and curcumin (CUR) were purchased from Shanghai Aladdin Biochemical Technology Co., Ltd. (Shanghai, China).

All other chemicals and solvents were of analytical grade and were used without further purification.

### 2.2. Cell Lines

Caco-2 cells were purchased from the Cell Bank of the Chinese Academy of Sciences (Shanghai, China) and cultured in MEM medium supplemented with 20% (*v*/*v*) fetal bovine serum, 100 U/mL penicillin, and 100 μg/mL streptomycin. Cultures were incubated at 37 °C in a humidified 5% CO_2_ environment.

HSC-T6 cells were purchased from the Cell Bank of the Chinese Academy of Sciences (Shanghai, China) and were cultured in DMEM medium supplemented with 10% (*v*/*v*) fetal bovine serum, 100 U/mL penicillin, and 100 μg/mL streptomycin. Cultures were incubated at 37 °C in a humidified 5% CO_2_ environment.

### 2.3. Animals

Male Sprague-Dawley (SD) rats (6–8 weeks; 220 ± 20 g; SPF) and male C57BL/6 mice (6–8 weeks; 20 ± 2 g; SPF) were purchased from Hunan Slack Jingda Experimental Animal Co., Ltd., Changsha, China. Experimental procedures on animals were undertaken following the National Guidelines and under the approval of the Animal Ethics Committee of Guilin Medical University for project (GLMC-IACUC-20251008).

### 2.4. Synthesis of Polymers and Preparation of Micelles

#### 2.4.1. Synthesis of Polymers and Preparation of Copolymer Micelles

ART, EDCI and DMAP (molar ratio 1:2:2) were weighed and dissolved in an appropriate amount of DMSO, then activated by stirring in a 60 °C–water bath for 1 h. COS was weighed and dissolved in pure water, then maintained in a constant temperature of 60 °C. The aqueous phase was slowly added to the organic phase and stirred in a 60 °C–water bath for 8 h. After cooling to room temperature, the solution was added to a dialysis bag (MWCO: 500 Da) and dialyzed for 24 h. The sample solution was concentrated through a cellulose membrane (MWCO: 10 kDa); then the concentrated solution was centrifuged at 8000 r/min for 10 min. The supernatant was filtered through a 0.45 μm filter membrane to obtain the ART–COS copolymer micelles solution (CA). The CA solution was freeze-dried and stored for later use.

RA, EDCI and NHS (molar ratio 1:1.2:1.2) were weighed and dissolved in an appropriate amount of DMSO, then activated by stirring in a 60 °C–water bath for 1 h and cooled to room temperature to obtain the RA solution. The pH of the ART–COS solution was adjusted to 9, then the mixture was slowly added to the RA solution and reacted at room temperature for 24 h. Then the solution was added to a dialysis bag (MWCO: 500 Da) and dialyzed for 24 h. The sample solution was filtered six times through a cellulose membrane (MWCO: 10 kDa); then the concentrated solution was centrifuged at 8000 r/min for 10 min. The supernatant was filtered through a 0.45 μm filter membrane to obtain a yellow sample solution, which is the RA-functionalized ART–COS copolymer micelles solution (RCA). The RCA solution was freeze-dried.

The molecular structure of CA and RCA was confirmed by ^1^H NMR (AVANCE NEO 400 MHz, Switzerland) and FTIR (Thermo Scientific iN10, USA). ART, RA and RCA were dissolved in d6-DMSO and COS in D_2_O for ^1^H NMR measurements. The FTIR spectra of these materials were recorded in the region of 400–4000 cm^−1^ in KBr tablets.

#### 2.4.2. Preparation of CUR-Loaded Micelles

The prescribed amount of CUR was weighed and dissolved in DMSO, then slowly added to the CA or RCA solution. The solution was sonicated in a water bath for 20 min and stirred magnetically at 300 r/min for 24 h. The sample solution was added to a dialysis bag (MWCO: 8–14 kDa), dialyzed overnight, and then centrifuged at 4000 r/min for 10 min. After filtered through a cellulose membrane (MWCO: 10 kDa), the concentrated solution was centrifuged at 8000 r/min for 10 min. The supernatant was filtered through a 0.45 μm filter membrane to obtain the nanomicelle solution (CUR@CA or CUR@RCA).

### 2.5. Characterization of Micelles

#### 2.5.1. Morphological Analysis

The micellar solution was diluted and dripped onto the carbon film supported by the copper grid and air-dried at ambient temperature prior to characterization. The morphological characteristics of RCA and CUR@RCA were then investigated using a transmission electron microscope (TEM, HT7700, Hitachi, Tokyo, Japan).

#### 2.5.2. Particle Size and Zeta Potential

The particle size distribution and zeta potential of RCA and CUR@RCA were determined using a particle size analyzer (Nano-ZS90, Malvern Instruments Ltd., Malvern, UK). Triplicate measurements were performed for each sample, and the mean values were calculated accordingly.

#### 2.5.3. Drug Loading Capacity

The drug loading capacity (DLC) of CUR@RCA was measured using a fluorescence spectrophotometer (RF-6000, Shimadzu, Kyoto, Japan). The setting condition of the fluorescence spectrophotometer were as follows: Ex = 430 nm; Em = 550 nm.

The DLC of the micelle can be calculated as follows:

DLC (%) = [Weight of CUR in micelles/(Weight of CUR@RCA)] × 100%.

#### 2.5.4. X-Ray Diffraction (XRD)

The polymorphic status of CUR encapsulated in the micelles was investigated using an X-ray diffractometer (Ultima IV, Rigaku, Akishima, Tokyo, Japan). The micellar suspension was freeze-dried, and the lyophilized powder was collected for subsequent analysis. The samples, including CUR; a simple physical mixture of ART, COS, RA, and CUR; RCA; and CUR@RCA, were scanned over the range of 5–60° at a scanning rate of 5°/min.

### 2.6. Critical Micelle Concentration (CMC)

The CMC values of CA and RCA were determined using the fluorescent probe method. A total of 0.05 mL of pyrene-in-methanol solution (0.012 mg/mL) was added into each test tube, respectively. The methanol in the solution was evaporated under a gentle stream of nitrogen. A series of CA and RCA solutions were added into each test tube, respectively. The CA solution with different concentrations (1, 5, 10, 25, 50, 100, 200, 300, 400, 500 μg/mL) and RCA solutions with different concentrations (1, 5, 10, 25, 50, 100, 150, 200, 250, 300 μg/mL) were prepared under ultrasonication for 40 min. The resulting solutions were equilibrated overnight in the dark at ambient temperature. Fluorescence intensities were recorded using a fluorescence spectrophotometer (RF-6000, Shimadzu, Kyoto, Japan) at an excitation wavelength of 334 nm, with emission wavelengths monitored at 373 nm (*I*_1_) and 393 nm (*I*_3_), respectively. The intensity ratio (*I*_3_/*I*_1_) was subsequently calculated.

### 2.7. In Vitro Release of Curcumin

The in vitro release profiles of CUR-loaded micelles were investigated in simulated gastric fluid (SGF), simulated intestinal fluid (SIF) and phosphate-buffered saline (PBS, pH 7.4) using a dialysis method. A free CUR solution was employed as the control. Accurate 2 mL amounts of CUR solution, CUR@CA, and CUR@RCA micellar solutions, equivalent to 168 μg/mL of CUR, respectively, were placed in a dialysis bag (MWCO = 1000 Da) against 20 mL of SGF (pH 2.0, containing 1% Tween 80), SIF (pH 6.8, containing 1% Tween 80) and PBS (pH 7.4, containing 1% Tween 80) as release mediums in a shaking incubator in the dark at 37 °C [56]. Samples (1 mL) were withdrawn at predetermined time intervals (0, 0.25, 0.5, 1, and 2 h for SGF; 0, 0.5, 1, 2, 4, 6 and 8 h for SIF; 0, 0.5, 1, 2, 4, 6, 8, 10, 12, 24, 36, 48, 60, and 72 h for PBS), and the content of CUR in the medium was determined by a fluorescence microplate reader (Infinite M200 Pro NanoQuant, Tecan Co. Ltd., Switzerland) at an excitation wavelength of 430 nm and an emission wavelength of 550 nm. The cumulative release percentage was determined, with all experiments conducted in triplicate.

### 2.8. Stability Studies

#### 2.8.1. Storage Stability

The prepared RCA and CUR@RCA micellar solutions were placed in bottles and stored in a refrigerator at 4 °C away from light for 4 week. The particle sizes and PDI values were respectively determined at predetermined intervals (1, 3, 5, 7, 14, 21 and 28 d), and the changes were observed.

#### 2.8.2. pH Stability

The RCA and CUR@RCA micellar solutions were diluted five times with SGF (pH 1.2), SIF (pH 6.8) and PBS (pH 7.4), respectively, and incubated at 37 °C. The particle size and PDI values were determined at predetermined intervals (0, 1, 2 and 4 h for SGF; 0, 2, 4, 6 and 8 h for SIF; 0, 2, 4, 6, 8, 10, 12, 24, 36, 48, 60, 72 h for PBS), and the changes were observed.

### 2.9. Pharmacokinetic Studies

Fifteen male SD rats weighing (200 ± 20) g were used in this animal experiment and were randomly divided into three groups (n = 5 per group). Prior to the experiment, all animals were fasted for 12 h with free access to water. The three groups were intragastrically administered CUR solution, CUR@CA, and CUR@RCA micelle solutions, respectively, at a CUR-equivalent dose of 15 mg/kg. Blood samples (0.3 mL) were collected from the caudal vein into heparinized tubes at predetermined time points (0.25, 0.5, 1, 1.5, 2, 4, 8, 12, and 24 h). Plasma was immediately obtained by centrifugation at 3500 rpm for 10 min. A 50 μL aliquot of plasma was transferred into a centrifuge tube, mixed with 200 μL of methanol, vortexed, and centrifuged at 12,000 rpm for 10 min. The content of CUR in the supernatant was determined using a fluorescence microplate reader (Infinite M200 Pro NanoQuant, Tecan Co. Ltd., Männedorf, Switzerland) (Ex = 430 nm; Em = 550 nm).

### 2.10. Oral Absorption Evaluation of Micelles

#### 2.10.1. Cell Viability Assay

The cytotoxicity of the micelle material was evaluated using the CCK-8 method. Approximate 1 × 10^4^ Caco-2 cells per well were seeded in a 96-well plate and cultured in 5% CO_2_ at 37 °C for 24 h. The culture medium was discarded from each well, and 100 μL of MEM supplemented with 20% FBS containing CA, RCA, CUR@CA, or CUR@RCA was added to the corresponding wells, followed by incubation for 24 h. According to the manufacturer’s instructions for the CCK-8 assay, 100 μL of working CCK-8 solution (CCK-8:MEM = 1:9, *v*/*v*) was added to each well and incubated for 2 h. The absorbance in each well was then measured using a microplate reader (EPOCH2, BioTek Instruments, Inc., Winooski, VT, USA) at 450 nm. Cell survival rate (%) is calculated according to the instructions and the measured absorbance. The absorbance of the cell-inoculated wells at 0 μg/mL is used as a negative control, and the absorbance of the pure culture medium without cells is used as a blank control. The calculation of cell survival rate is as follows:

Cell survival rate (%) = (OD_sample_ − OD_blank sample_)/(OD_control_ − OD_blank sample_) × 100%

#### 2.10.2. Cellular Uptake Assay

Caco-2 cells were seeded into 12-well plates at a density of approximate 1 × 10^5^ cells per well and cultured in 5% CO_2_ at 37 °C for 24 h. Then the cells were treated with CUR@CA and CUR@RCA, respectively, in solution in 1 mL of serum-free medium per well. After 2, 3, and 4 h of incubation, the cells were trypsinized, centrifuged at 1800 rpm and 4 °C for 5 min, collected, and suspended in PBS. The percentage of fluorescence-positive cells was measured by flow cytometry (BD AccuriTM C6 Plus, BD Biosciences, San Jose, CA, USA).

To detect CUR@CA and CUR@RCA using an inverted fluorescence microscope (OLYMPUS, Tokyo, Japan), cells were seeded onto coverslips in culture plates, incubated for 24 h, then treated for 3 h with CUR@CA or CUR@RCA. Cells were rinsed with ice-cold PBS, fixed in 4% paraformaldehyde at room temperature for 15 min, and then stained with DAPI for 10 min to visualize the nuclei. Samples were observed using an inverted fluorescence microscope (OLYMPUS, Tokyo, Japan).

#### 2.10.3. Cellular Uptake Mechanism

Caco-2 cells were seeded into the 24-well plate at a density of approximate 5 × 10^4^ cells per well and cultured in 5% CO_2_ at 37 °C for 24 h. The culture media were replaced with 1 mL of inhibitor-in-HBSS solutions, including colchicine (5 μM), sodium azide (46 mM), indomethacin (200 μM), chlorpromazine (28 μM), quercetin (10 μM), and retinoic acid (20 μM), for a 30 min pre-incubation at 37 °C. The CUR@RCA micelle solutions were added into the respective inhibitor solutions up to 5.25 μg/mL of CUR for further 2 h incubation. The follow-up operation was the same as that outlined in the previous section. The percentage of fluorescence-positive cells was measured by flow cytometry (BD AccuriTM C6 Plus, BD Biosciences, San Jose, CA, USA).

#### 2.10.4. Transport Through Caco-2 Cells Monolayers

Prior to the transport study, Caco-2 cells seeded in the Transwell chambers were washed three times with HBSS and equilibrated in HBSS at 37 °C for 30 min. Subsequently, 0.3 mL of CUR@CA and CUR@RCA micelle solutions (with CUR concentration adjusted to 1.8 μg/mL) were added to the apical (AP) chambers. At predetermined time points (0.5, 1, 1.5, 2, and 3 h), 0.2 mL aliquots were collected from the basolateral (BL) chambers, and an equal volume of fresh HBSS was replenished immediately. The samples were subsequently analyzed using a fluorescence microplate reader (Infinite M200 Pro NanoQuant, Tecan Co. Ltd., Männedorf, Switzerland) (Ex = 430 nm; Em = 550 nm). The apparent permeability coefficients (*P*_app_, cm/s) of CUR were calculated as follows.

$$P_{app}=\frac{Q}{A\times C\times T}$$

*Q*: The total quantity of CUR permeated (ng);

*A*: The surface area of the apical chamber of cell monolayers (cm^2^);

*C*: The initial quality of CUR in the apical chamber (ng/cm^3^);

*T*: The incubation time of the administration (h)

#### 2.10.5. Transporter Immunofluorescence Localization

Caco-2 cells were seeded onto the sterilized coverslips placed in the 24-well plate at a density of 5 × 10^4^ cells per well and cultured overnight in 5% CO_2_ at 37 °C. The medium was replaced with serum-free MEM, and then the CUR@CA and CUR@RCA micelle solutions, containing 20 μg/mL of CUR, were each added to the wells for 2 h uptake incubation. Cells were rinsed with ice-cold PBS and fixed in 4% paraformaldehyde at room temperature for 20 min.

Immunofluorescence staining of cellular tight junction protein was performed according to the manufacturer’s instructions. Briefly, cells were incubated with anti-ZO-1 rabbit polyclonal antibody (21773-1-AP, Proteintech, Wuhan, China, 1:1000) as the primary antibody, followed by CoraLite594-conjugated goat anti-rabbit IgG (SA00013-4, Proteintech, 1:500) as the secondary antibody. Subsequent procedures for DAPI staining and slide preparation were identical to those described in a previous section of this paper. The interaction between the target micelles and ZO-1 was visualized using an inverted fluorescence microscope (OLYMPUS, Tokyo, Japan).

### 2.11. In Vitro Efficacy Evaluation of Micelles

#### 2.11.1. Cell Viability Assay of Activated HSC-T6

Approximately 1 × 10^4^ HSC-T6 cells per well were seeded in a 96-well plate and cultured in 5% CO_2_ at 37 °C for 24 h. Then the cells were activated by TGF-β1 (10 ng/mL). After a 24 h incubation, the culture medium was removed from each well, and 100 μL of DMEM supplemented with 10% FBS containing CA, RCA, CUR@CA, or CUR@RCA was added to the corresponding wells, followed by another 24 h incubation. Subsequently, 100 μL of CCK-8 working solution (CCK-8: DMEM = 1:9, *v*/*v*) was added to each well and incubated for 2 h, according to the manufacturer’s instructions. The absorbance of each well was then measured using a microplate reader (EPOCH2, BioTek Instruments, Inc., Winooski, VT, USA) at 450 nm. Cell survival rate (%) is calculated according to the instructions and the measured absorbance. The absorbance of the cell-inoculated wells at 0 μg/mL is used as a negative control, and the absorbance of the pure culture medium without cells is used as a blank control. The calculation of cell survival rate is as follows:

Cell survival rate (%) = (OD_sample_ − OD_blank sample_)/(OD_control_ − OD_blank sample_) × 100%

#### 2.11.2. Cellular Uptake of Activated HSC-T6

HSC-T6 cells were seeded into 12-well plates at a density of approximate 1 × 10^5^ cells per well and cultured in 5% CO_2_ at 37 °C for 24 h. Then cells were activated by TGF-β1 (10 ng/mL). After 24 h, activated cells were treated with CUR solution, CUR@CA and CUR@RCA micelle solutions, respectively, in 1 mL of serum-free medium per well. After 1, 2, 4, and 6 h of incubation, cells were trypsinized, centrifuged at 1800 rpm and 4 °C for 5 min, collected, and suspended in PBS. The percentage of fluorescence-positive cells was measured by flow cytometry (BD AccuriTM C6 Plus, BD Biosciences, San Jose, CA, USA).

To detect CUR, CUR@CA and CUR@RCA using an inverted fluorescence microscope, cells were seeded onto coverslips in culture plates, incubated for 24 h, then activated by TGF-β1 (10 ng/mL) for 24 h and treated for 2 h with CUR, CUR@CA or CUR@RCA. Cells were washed with cold PBS and fixed with 4% paraformaldehyde at room temperature for 15 min; then nuclei were stained for 10 min with DAPI. Samples were observed using a laser scanning confocal microscope (Olympus FV3000, Hachioji, Tokyo, Japan).

#### 2.11.3. Lipid ROS Detection

HSC-T6 cells were seeded into 12-well plates at a density of approximate 1 × 10^5^ cells per well and cultured in 5% CO_2_ at 37 °C for 24 h. Then cells were activated by TGF-β1 (10 ng/mL). After 24 h, the medium was removed from each well, and an aliquot of 1 mL DMEM (10% FBS) containing CUR, CA, RCA, CUR@CA and CUR@RCA were added, respectively, followed by incubation for 24 h. After incubation for 20 min using a DCFH-DA fluorescent probe, cells were trypsinized, centrifuged at 1800 rpm and 4 °C for 5 min, collected, and suspended in PBS. The percentage of fluorescence-positive cells was measured by flow cytometry (BD AccuriTM C6 Plus, BD Biosciences, San Jose, CA, USA).

#### 2.11.4. Real-Time qPCR Analysis

The process of cell culture, activation and drug administration was the same as that used in the above section. After 24 h of drug administration, the culture medium was removed, and the cells were washed three times with PBS buffer. Total cellular RNA was extracted using the TRIzol reagent. RNA content was measured using an ultra-micro nucleic acid protein concentration analyzer (Thermo Scientific, Waltham, MA, USA). The RNA was reverse transcribed into cDNA using the ToloScript All-in-one RT EasyMix for qPCR kit (TOLOBIO, Shanghai, China). RT–qPCR was conducted using the 2 × Q3 SYBR qPCR Master Mix (Universal) kit (TOLOBIO, Shanghai, China) via a real-time fluorescence quantitative PCR instrument QuantStudio 3 (Thermo Scientific, Waltham, MA, USA). Results were normalized to GAPDH. Primer sequences are as follow: (Ferritin: Forward 5′-AACCTGACCGTGATGACT-3′, Reverse 5′-GGTAATGCGTCTCAATGAAG-3′, GPX4: Forward 5′-CAGGAGCCAGGAAGTAATC-3′, Reverse 5′-GCAGCCGTTCTTATCAATG-3′, CL: Forward 5′-CCTGCTTGCTATGTCCTTA-3′, Reverse 5′-AGTGTCTTGTCTCCTGGTA-3′, GAPDH: Forward 5′-TCTCCTGCGACTTCAACA-3′, Reverse 5′-TGTAGCCGTATTCATTGTCA-3′).

#### 2.11.5. Western Blot Analysis

HSC-T6 cells were treated in the indicated ways, harvested, and lysed in lysis buffer supplemented with phenylmethyl sulfonyl fluoride. Sampling protein solution containing 20% SDS, phosphate buffer, and lysate was fractionated on a 10% SDS-polyacrylamide gel (Epizyme, Shanghai, China), blotted onto a polyvinylidene fluoride membrane (0.45 μm, Millipore, Burlington, MA, USA), and blocked for 2 h with 5% milk in Tris-buffered saline containing 0.1% Tween-20. Blots were then incubated with primary antibody against GPX4 (Servicebio, China) overnight at 4 °C, washed, incubated with appropriate secondary horseradish peroxidase-conjugated secondary antibody (Servicebio, China) for 1 h at room temperature, and developed using a chemiluminescent substrate (Epizyme, Shanghai, China). Blot testing was was performed in quintuplicate and quantified by densitometry using Image J software (Version 1.53k, National Institutes of Health, Bethesda, MD, USA).

### 2.12. In Vivo Pharmacodynamic Evaluation of Micelles

#### 2.12.1. Establishment of Hepatic Fibrosis Model

The male C57BL/6 mice used for the CCl_4_-induced hepatic fibrosis model were randomly divided into groups and provided with free access to water and laboratory chow. Fibrosis model mice received intraperitoneal injections of CCl_4_ in olive oil (20%, 5 mL/kg body weight) twice weekly for 4 weeks, whereas mice in the control group received olive oil intraperitoneally.

#### 2.12.2. Assay of Anti-Fibrosis Efficiency In Vivo

After 4 weeks of modeling, C57BL/6 mice were randomly divided into six groups (*n* = 8) and were administered with a twice-weekly gastric gavage of saline, free ART&CUR, CA, RCA, CUR@CA or CUR@RCA (ART equivalent: 10 mg/kg) for 4 weeks. Other mice injected intraperitoneally with olive oil and orally administered with saline served as healthy controls. After 4 weeks of the treatment, the mice were sacrificed, and both serum and major organs were collected. Hepatic fibrosis was assessed using hematoxylin–eosin (H&E) and Masson staining. Serum alanine transaminase (ALT) and aspartate aminotransferase (AST) levels were measured using kits. Collagen I, GPX4, MMP-1, and TIMP-1 levels in liver tissues were examined through Western blotting, while α-SMA levels were analyzed through immunofluorescence.

### 2.13. Statistical Analysis

All data are presented as mean ± standard error of mean (SEM). Statistical analysis was performed using ANOVA in GraphPad Prism 8.0 software (San Diego, CA, USA). Statistical significance was set at *p* < 0.05 (with ^#^ *p* < 0.05; ^##^ *p* < 0.01; ^###^ *p* < 0.001; ^####^ *p* < 0.0001; * *p* < 0.05; ** *p* < 0.01; *** *p* < 0.001; **** *p* < 0.0001).

## 3. Results and Discussions

### 3.1. Characterization of Polymers (CA and RCA)

**Figure 1:**
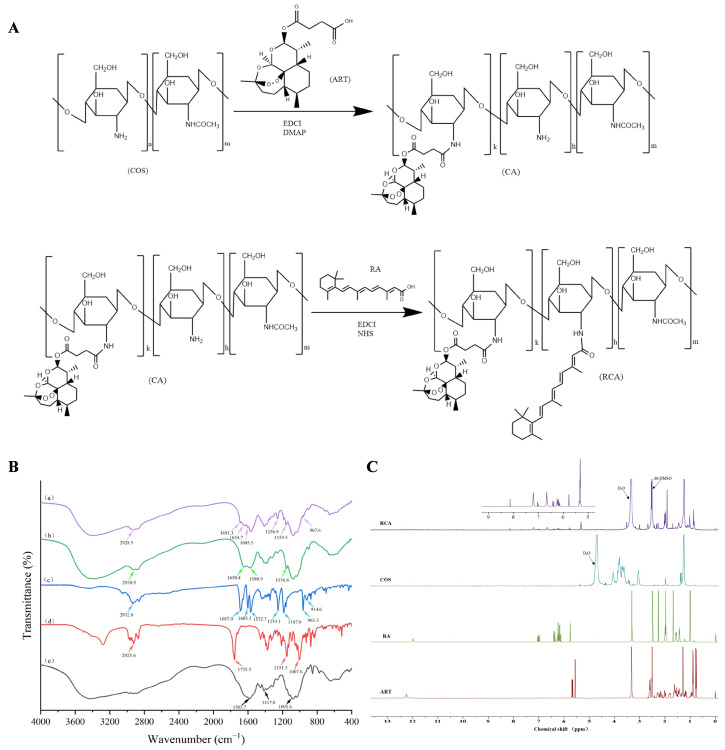
Synthesis and characterization of polymers. (**A**) Synthetic route of CA and RCA. (**B**) FTIR spectra of CA and RCA, (a) RCA, (b) CA, (c) RA, (d) ART, and (e) COS. (**C**) ^1^H NMR spectra of RCA.

The micelle skeleton of CA and RCA was synthesized by EDCI-mediated amido formation reaction, as shown in Figure 1A. The carboxyl group of ART was activated by EDCI and facilely conjugated to the amine group of COS. After synthesis of CA, the carboxyl group of RA activated by EDCI was conjugated to the amine group of CA. During the dialysis process, CA and RCA micelles were formed through self-assembly.

The molecular structure of the synthesized conjugates was characterized by FTIR and ^1^H NMR. The FTIR spectra of RA, ART, COS, CA, and RCA are displayed in Figure 1B. In the COS spectrum, characteristic absorption bands were observed at 3400 cm^−1^, 1585 cm^−1^, and 1417 cm^−1^, corresponding to the overlapped stretching vibrations of -OH and -NH_2_, the C–H bending of -CH_3_, and the N–H deformation vibration of the amino groups, respectively. The typical COS signal at 1091 cm^−1^, assigned to the C–O stretching vibration, was also retained in the CA spectrum. Compared with COS, the CA conjugate exhibited an enhanced absorption peak at approximately 1650 cm^−1^, attributed to the C=O stretching vibration of secondary amides, together with a peak at 1580 cm^−1^ corresponding to the C–N stretching vibration. Meanwhile, the characteristic ART band at 1156 cm^−1^, ascribed to the ester bond, was also detected in CA. These results collectively confirmed the formation of an amide linkage between the carboxyl groups (-COOH) of ART and the amino groups (-NH_2_) of COS, verifying the successful conjugation of ART to COS. Compared to the CA spectrum, the several typical absorption peaks of RA, including 1691 cm^−1^, 1605 cm^−1^, 967 cm^−1^, and 1256 cm^−1^ assigned to the stretching vibration and bending vibration of C=C, and the vibration peak of C-H on the aromatic ring were presented in the spectrum of RCA. The results indicated that RA was successfully conjugated to CA.

The chemical structure of the as-synthesized RCA was further confirmed by ^1^H NMR, as shown in Figure 1C. The chemical shift of ART at 5.5–5.7 ppm was assigned to methylene hydrogen between the peroxide bridge and the epoxy group. The chemical shift of COS at 1.2 ppm was assigned to methyl hydrogen of the acetamido group, and the multi-peaks of COS at 3.0–3.5 ppm were its characteristic chemical shifts. The chemical shift of RA at 1.0 ppm was assigned to aliphatic chain methyl hydrogen, and the chemical shifts of RA at 2.0 ppm and 5.7–7.1 ppm were assigned to conjugated double-bond hydrogen. The carboxyl hydrogen signal peak of ART and RA at 12–12.5 ppm disappeared in RCA, and the new hydrogen peak on the amide bond at 6.6–7.2 ppm, as well as the above ART, COS, and RA characteristic peaks, were retained in RCA, indicating that the RCA synthetic route was based on the combination reaction of ART, COS and RA. The grafting ratio of ART and RA was 23.81% and 7.08%, respectively, as calculated using ^1^H NMR.

### 3.2. Preparation and Characterization of Micelles

**Figure 2:**
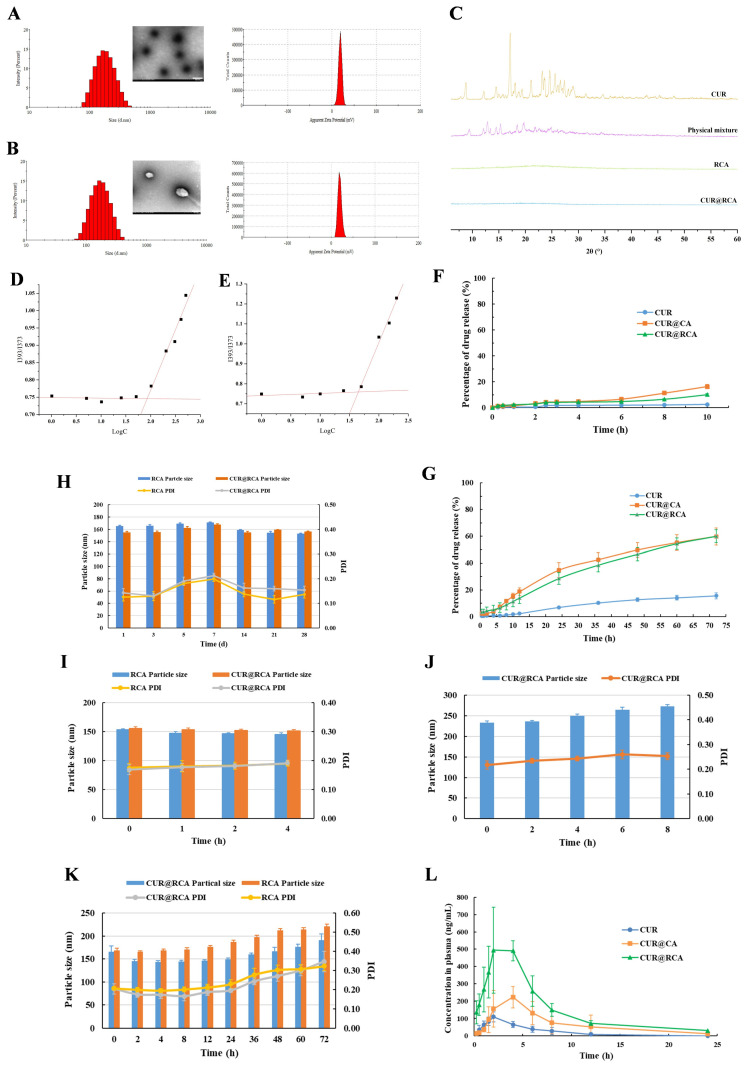
Characterization of micelles. Particle size distribution, zeta potential and TEM image of RCA (**A**) and CUR@RCA (**B**). (**C**) The XRD curves of CUR, the physical mixture of CUR and RCA, and CUR@RCA. The curve of the fluorescence intensity ratio (I_393_/I_373_) from pyrene vs. the logarithmic concentration of the material CA (**D**) and RCA (**E**). (**F**) The in vitro release profiles of CUR, CUR@CA and CUR@RCA in simulated gastric fluid (0–2 h) and simulated intestinal fluid (2–10 h). (**G**) The in vitro release profiles of CUR, CUR@CA and CUR@RCA in phosphate buffer (0–72 h). (**H**) The stability of micellar solutions stored at 4 °C for 28 days (n = 3). (**I**–**K**) The stability of micellar solutions in SGF (pH 2.0) over 4 h, SIF (pH 6.8) over 8 h and in PBS buffer (pH 7.4) over 72 h (n = 3). (**L**) Oral pharmacokinetic profiles of CUR, CUR@CA, and CUR@RCA in rats, as per the CUR-equivalent dose of 15 mg/kg. The data are expressed as means ± SD (n = 5).

Owing to the self-assembly of amphiphilic RCA, the model drug CUR was efficiently encapsulated into the micelles. As shown in Figure 2A,B, the average particle size of the RCA and CUR@RCA micelles were, respectively, (165.27 ± 1.33) nm and (155.90 ± 0.78) nm, and the polydispersity index (PDI) was lower than 0.2, which indicates that the micelles have good monodispersity. The results were consistent with those observed by transmission electron microscopy (TEM) (Figure 2A,B). TEM images showed that the micelles had a nearly spherical structure, which further confirmed their homogeneity. Owing to the abundant amino groups of COS, the micelles displayed positive zeta potentials of (19.27 ± 0.45) mV and (18.73 ± 0.45) mV, which facilitated adhesion to the negatively charged intestinal epithelial cell membrane and consequently, enhanced the oral absorption of the loaded CUR. The DLC of the CUR@RCA micelles was (1.78 ± 0.05) %, which was mainly attributed to the fact that rigid molecular steric hindrance greatly limits further drug loading efficiency.

The crystallization behavior of CUR in the micelles was investigated by XRD. The XRD spectra of CUR, a simple physical mixture containing ART, COS, RA and COS, lyophilized RCA and CUR@RCA, as shown in Figure 2C. The CUR was highly crystalline, whereas the RCA was amorphous. As depicted in Figure 2C, the characteristic diffraction peaks of CUR in the range from 10° to 30° (2θ) were clearly observed in the physical mixture. In contrast, these crystalline diffraction signals of CUR were absent in the diffractogram of the CUR@RCA micelles, suggesting that CUR encapsulated in the micelles existed in a molecularly dispersed or amorphous state rather than in crystalline form.

### 3.3. Critical Micelle Concentration (CMC)

CMC, a vital indicator for assessing self-aggregation propensity, was determined to characterize the micellization process. The aggregation behaviors of CA and RCA in aqueous media were examined by fluorescence probe spectroscopy, with pyrene as the probe molecule. The curve of I_393_/I_373_ values vs. the logarithmic concentration of the micelle material CA and RCA are shown in Figure 2D,E. There was not a significant change in the ratio value (I_393_/I_373_) in the range of low concentration, indicating that the solution polarity was slowly changed by the addition of CA and RCA. Upon reaching a specific concentration, the ratio exhibited a sharp increase in its growth rate. The concentration corresponding to this transition point was defined as CMC, indicating the onset of micellization and the subsequent incorporation of pyrene into the micellar core. The CMC values, determined from the intersection of the fitted linear segments, were measured to be 85.25 μg/mL for CA and 44.98 μg/mL for RCA. These relatively low CMC values demonstrate that both CA and RCA can readily self-assemble into stable micelles, even at low concentrations in aqueous media. Accordingly, such self-assembled micelles possess excellent anti-dilution stability against gastrointestinal fluid, enabling them to maintain their intact micellar structure during intestinal transit and preserve the encapsulated drug.

### 3.4. In Vitro Release of Curcumin

In vitro release of CUR from the micelles was performed by dialysis in SGF, SIF, and PBS (pH 7.4), as illustrated in Figure 2F,G. Obvious discrepancies in release profiles were observed in different media. In SGF and SIF, the percentage of CUR release was about 10% over 10 h. However, in PBS, an extremely sustained release, conforming to the zero-order function (*Q* = 3.41365 + 0.85408 *t*, *R*^2^ = 0.9836), was observed in the CUR@RCA, with about a 60% drug release over 72 h. The distinct difference in drug release profiles suggested that CUR@RCA micelles were capable of preventing premature drug leakage in the intestinal lumen prior to cellular internalization into enterocytes. While the micelles absorbed into the blood could release the drug at a constant rate, prolonging the circulation time of the drug in the blood to exerted its targeted action.

### 3.5. Stability of Micelles

The results of stability studies were shown in Figure 2H–K. As shown in Figure 2H, the significant variation in the particle size and PDI of micelles in solution was not found within 4 week when stored in a refrigerator at 4 °C away from light (RSD < 5%). The micelles can be stored at 4 °C for about one month, which is convenient for carrying out relevant experimental research. As shown in Figure 2I, the particle size and PDI of both the RCA and CUR@RCA micelles remained almost unchanged (RSD < 3%) after 4 h in SGF (pH 2.0), indicating good stability of the two micelle solutions in gastric fluid. As shown in Figure 2J, the particle size and PDI of the CUR@RCA micelles also remained almost unchanged (RSD < 7%) after 8 h in SIF (pH 6.8). However, it was observed during the experiment that the particle size and PDI of the RCA micelles increased significantly and precipitated after dilution with SIF, indicating that the RCA micelles were unstable in intestinal fluid. The instability was improved after CUR was loaded into the RCA micelles.

As shown in Figure 2K, the particle size and PDI of the CUR@RCA micelles remained almost unchanged after 48 h in PBS (pH 7.4), with a slight increase after 48 h (RSD < 10%). The particle size and PDI of the RCA micelles remained almost unchanged after 24 h in PBS buffer (pH 7.4), and began to increase after 24 h (RSD < 12%). These results indicated that the CUR@RCA micelles had good stability in the gastrointestinal tract and blood, suggesting that the CUR@RCA micelles can carry drugs to target hepatic stellate cells and then exert therapeutic effects.

### 3.6. Pharmacokinetic Evaluation

The oral pharmacokinetic study of CUR@CA and CUR@RCA was carried out on SD rats against CUR as a reference formulation. The fluorescence method was applied for curcumin quantification [57]. Method validation, including specificity, linearity, accuracy, precision and recovery, was carried out, and all indices satisfied the criteria for biological sample detection. The regression equation was F = 282.59C + 13.166 (R^2^ = 0.9973), and CUR exhibited favorable linearity at concentrations from 0.103 to 1.03 μg/mL. The RSD values for precision, repeatability and recovery were 2.93%, 4.21% and 6.17% (recovery: 90–110%), respectively. The pharmacokinetic parameters are listed in Table 1, and the curves of the drug concentration in plasma vs. time are shown in Figure 2L. It was observed that the CUR@CA and CUR@RCA manifested a higher C_max_ than did CUR. Compared to the CUR solution, the peak concentration (*C*_max_) of CUR@CA and CUR@RCA were 2.03-fold and 4.52-fold, respectively, and the oral relative bioavailability (*Fr*) of CUR@CA and CUR@RCA were 298.39% and 659.72%, respectively, calculated by the ratio of *AUC*_(0−t)_, indicating enhanced oral absorption. It was worth noting that the addition of RA increased the *C*_max_ and *Fr* of CUR@RCA and produced an absorption enhancement of CUR in the micelles. It is preliminarily speculated that due to retinoic acid receptors existing on intestinal epithelial cells, retinoic acid on the micelles could interact with these receptors, increasing the cellular affinity and penetration of the micelles. This interaction may promote the internalization of micelles in intestinal epithelial cells, thereby promoting the oral absorption of drugs, which is consistent with the experimental results of the cell uptake mechanism. The CUR@RCA showed a lower apparent distribution volume (*V*_z_/*F*) than that of CUR, implying the reduced drug diffusion into peripheral tissues, increased blood retention and prolonged in vivo circulation time. Moreover, CUR in the micelles exhibited markedly sustained in vitro release, yet it surprisingly achieved superior oral bioavailability relative to the free CUR solution. These results imply that the CUR@RCA micelles were predominantly absorbed as intact nanostructures, rather than undergoing premature drug release in the gastrointestinal tract prior to enterocyte internalization. Compared with the CUR solution, the prolonged *MRT*_(0−t)_ and reduced *CL*_z_/*F* values observed for the micelle formulation indicated slower systemic elimination of the micelle-loaded drug.

### 3.7. Oral Absorption Evaluation of Micelles

#### 3.7.1. Cell Viability

**Figure 3:**
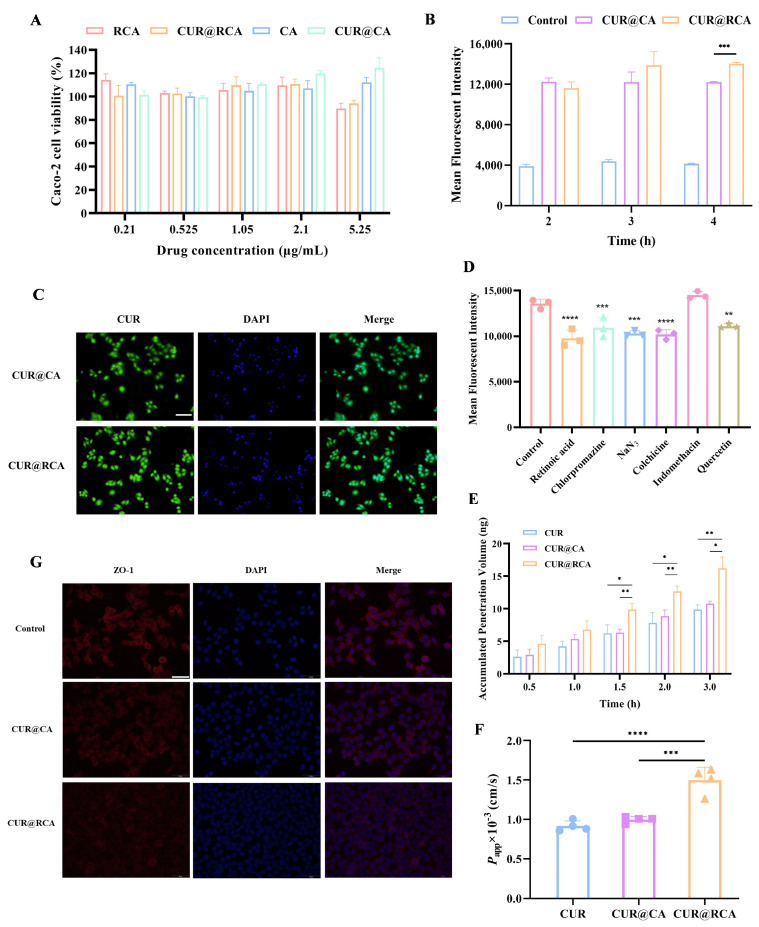
Oral absorption evaluation of micelles in vitro. (**A**) Caco-2 cell viability in the presence of CA, RCA, CUR@CA and CUR@RCA, as per the CUR-equivalent concentration in the x-axis, respectively. The data are expressed as means ± SD (n = 3). (**B**) The quantitative uptake histogram of CUR@CA and CUR@RCA in Caco-2 cells, with data presented as mean ± SD (n = 3). (**C**) The qualitative uptake of CUR@CA and CUR@RCA in Caco-2 cells. Scale bar = 100 μm. (**D**) The mean fluorescent intensity of CUR in CUR@RCA across Caco-2 in absence or presence of various endocytosis inhibitors, with data presented as mean ± SD (n = 3). (**E**) The accumulated penetration volume of CUR in CUR, CUR@CA and CUR@RCA across Caco-2 monolayer. Data are shown as mean ± SD (n = 4). (**F**) The apparent permeability coefficient (Papp) of CUR in CUR, CUR@CA and CUR@RCA across Caco-2 monolayer (n = 4). (**G**) The impact of CUR, CUR@CA and CUR@RCA on the tight junction protein ZO-1 in Caco-2 cells. Scale bar = 50 μm. All data are shown as mean ± SD (* *p* < 0.05; ** *p* < 0.01; *** *p* < 0.001; **** *p* < 0.0001).

The results of cell viability after the action of the micelle solutions of different concentrations on Caco-2 cells are shown in Figure 3A. The results indicated that within the measured concentration range, the viability of Caco-2 cells is greater than 80%. It suggested that the micelle solutions had no significant toxicity to Caco-2 cells. Therefore, the concentration of CUR within the range of 0.21 to 5.25 μg/mL could be used for experiments such as those evaluating cell uptake and transport.

#### 3.7.2. In Vitro Cellular Uptake Studies

The in vitro uptake of drug-loaded micelles was evaluated through the quantitative and qualitative uptake by Caco-2 cells. The quantitative uptake of drug-loaded micelles in Caco-2 cells at different times is shown in Figure 3B. The results indicated that the uptake of CUR@CA and CUR@RCA was time-dependent. Notably, the uptake of CUR@CA plateaus after 2 h, while the uptake of CUR@RCA continues to increase significantly at 3 h and was higher than that of CUR@CA, which was consistent with the qualitative uptake results. This preliminary evidence suggests that CUR@RCA can enhance intestinal absorption and is superior to CUR@CA. The qualitative uptake by Caco-2 cells is shown in Figure 3C. The results showed that after a 3 h incubation, CUR is primarily concentrated in the nucleus, indicating that the drug-loaded micelles can be taken up by Caco-2 cells. It is speculated that after the micelles enter the cells, they transport the drug through endocytosis.

#### 3.7.3. Transporter-Mediated Endocytosis

To explore the precise endocytic route of the CUR@RCA micelles, cellular uptake and transport across Caco-2 monolayers were assessed after pretreatment with various endocytosis inhibitors. As shown in Figure 3D, the endocytosis of CUR@RCA was inhibited in the presence of sodium azide (an energy inhibitor), chlorpromazine (a clathrin-mediated endocytosis inhibitor), colchicine (a macropinocytosis inhibitor), indomethacin (a caveolae-mediated endocytosis inhibitor), quercetin (a synaptic vesicle presynaptic inhibitor), and retinoic acid (a retinoic acid receptor inhibitor), respectively. Among these inhibitors, indomethacin showed no inhibitory effects on the endocytosis of CUR@RCA, and the mean fluorescent intensity of the micelles was reduced after the treatment with sodium azide, chlorpromazine, colchicine and quercetin, suggesting that the cellular endocytosis of the micelles was dependent on macropinocytosis and clathrin mediation with energy consumption. Compared to the control, the higher fluorescence intensity of CUR@RCA indicated the effective transportation capacity of the micelles. Furthermore, the lower fluorescence intensity of the micelles observed in the presence of free RA, compared with that in the absence of RA, provided additional evidence for the transporter-mediated endocytosis mechanism. This finding was consistent with the competitive inhibition results obtained from both pharmacokinetic and cellular uptake assays.

#### 3.7.4. Transport Through Caco-2 Cell Monolayers

It was reported that, when the TEER value was greater than 300 Ω/cm^2^, Caco-2 cells could be used to measure the penetration of drugs. The Caco-2 cell monolayer transport model was constructed in this study. The transport results of drug-loaded micelles through the Caco-2 cell monolayer are shown in Figure 3E,F. The penetration through the Caco-2 cell monolayer indicated that the transport of CUR@RCA was significantly increased as cultivating prolonged and higher than that of CUR and CUR@CA. The Papp value of CUR@RCA was about 1.5-fold higher than that of CUR and CUR@CA. It further confirmed that CUR@RCA could enhance drugs transport through the Caco-2 cell monolayer.

#### 3.7.5. Immunofluorescence Localization

Zonula occludens 1 (ZO-1) is one of the essential components of tight junctions, and the downregulation of its expression or reduction in activity can affect the formation of intercellular tight junctions, thereby affecting the defensive barrier function of the intestinal mucosa. This study investigated the impact of micelles on the tight junction protein ZO-1 in Caco-2 cells to explore the pathways of paracellular transport of micelles. The immunofluorescence results shown in Figure 3G indicated that the red fluorescence of the tight junction protein ZO-1 in the Caco-2 cell monolayers treated with CUR@CA and CUR@RCA micelles was weakened to varying degrees compared with that in CUR, with the CUR@RCA group showing a significant decrease in red fluorescence. The results indicated that the CUR@CA and CUR@RCA micelles had a certain inhibitory effect on the expression of ZO-1, with the CUR@RCA micelles having a stronger inhibitory effect. It is speculated that CUR@RCA can, to some extent, open the tight junctions between cell monolayers to increase the paracellular transport of drugs, thereby promoting oral absorption.

### 3.8. In Vitro Pharmacodynamic Evaluation of Micelles

#### 3.8.1. In Vitro Cell Viability and Proliferation Inhibition

**Figure 4:**
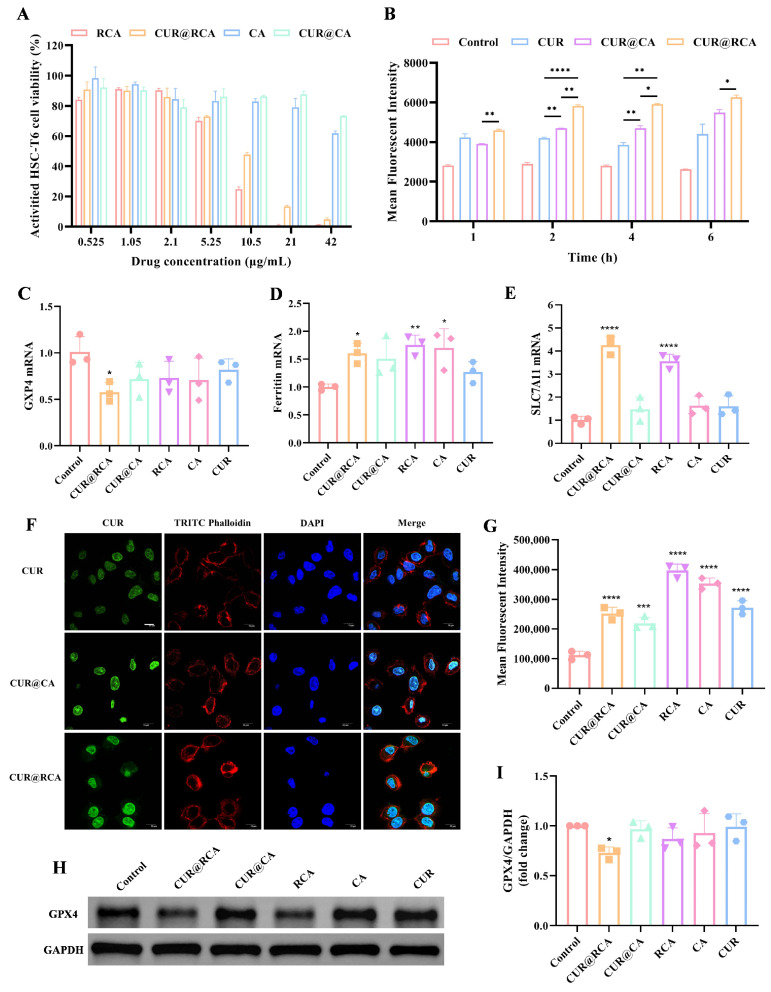
In vitro pharmacodynamic evaluation of micelles. (**A**) TGF-β1-activated HSC-T6 cell viability in the presence of CA, RCA, CUR@CA and CUR@RCA, as per the CUR-equivalent concentration in the x-axis, respectively. (**B**) The quantitative uptake histogram of CUR, CUR@CA and CUR@RCA in TGF-β1-activated HSC-T6 cells. (**C**–**E**) Real-time PCR analysis of GPX4, ferritin, and SLC7A11 mRNA levels in TGF-β1-activated HSC-T6 cells after treatment with various formulations. GAPDH was included as loading control. (**F**) The qualitative uptake of CUR, CUR@CA and CUR@RCA in TGF-β1-activated HSC-T6 cells. Scale bar = 10 μm. (**G**) The ROS levels in TGF-β1-activated HSC-T6 cells after treatment with various formulations. (**H**) Western blot analysis of GPX4 levels in TGF-β1-activated HSC-T6 cells after treatment with various formulations. GAPDH was included as loading control. (**I**) Quantitative analysis of GXP4 in activated HSC-T6 cells. All data are presented as mean ± SD (n = 3; * *p* < 0.05; ** *p* < 0.01; *** *p* < 0.001; **** *p* < 0.0001).

The results of the proliferation inhibition effect on TGF-β1-activated HSC-T6 cells by micelle solutions of different concentrations are shown in Figure 4A. As the drug concentration increased, the toxicity of CUR-loaded micelles to activated HSC-T6 cells gradually increased. RCA and CUR@RCA were more cytotoxic against activated HSC-T6 cells than were CA and CUR@CA at the same dose. These results suggest that the modification of RA significantly increases the accumulation concentration of micelles in activated HSC-T6 cells, thereby enhancing cytotoxicity.

#### 3.8.2. Cellular Uptake and Intracellular Distribution In Vitro

The in vitro uptake of drug-loaded micelles was evaluated through quantitative and qualitative uptake by TGF-β1-activated HSC-T6 cells, aiming to explore their in vitro targeting properties. The quantitative uptake of drug-loaded micelles in activated HSC-T6 cells at different times is shown in Figure 4B. The results indicated that the uptake of CUR@CA and CUR@RCA was time-dependent. Notably, after 2 h, the uptake of CUR-loaded micelles was significantly higher than that of CUR solutions. Furthermore, the uptake of CUR@RCA exceeded that of CUR@CA, which was consistent with the qualitative uptake results. This preliminary evidence suggested that CUR-loaded micelles can focus on activating HSC-T6 cells. The qualitative uptake by activated HSC-T6 cells is shown in Figure 4F. The results showed that after a 2 h incubation, CUR is primarily concentrated in the nucleus, indicating that the drug-loaded micelles can be taken up by activated HSC-T6 cells. It is speculated that after entering the cells, the micelles released the drug to exert therapeutic effects. Though the RA-mediated targeting effect was verified through cellular uptake and intracellular distribution results, more direct molecular validation is still required in future work.

#### 3.8.3. The Mechanism of Inhibiting HSCs Proliferation and Activation

Ferroptosis is a form of iron-dependent cell death. Recent studies have found that it is closely related to many diseases, such as hepatic fibrosis and cancer [58,59]. In this paper, we used RT–qPCR to detect the expression of GPX4, ferritin, and SLC7A11 mRNA in activated HSC-T6 cells, which are related to ferroptosis. The results are shown in Figure 4C–E. Compared with the control group, the micelle groups regulated the levels of GPX4, ferritin, and SLC7A11 mRNA to varying degrees. The expression of GPX4 mRNA was significantly decreased, while the expression of ferritin and SLC7A11 mRNA was significantly increased. It is speculated that the micelles may modulate ferroptosis in activated HSCs via regulating ferroptosis-related pathways. They may also suppress HSC activation through ferroptosis-associated changes and reduce ECM deposition, which helps attenuate hepatic fibrosis.

As an antioxidant enzyme, glutathione peroxidase (GPX4) is a key target for regulating ferroptosis. GPX4 can remove reactive oxygen species and inhibit lipid peroxidation, thereby protecting cells from ferroptosis. When the expression level of GPX4 decreases, It can modulate ferroptosis in activated HSCs, which is an effective strategy for treating hepatic fibrosis. Therefore, we used Western blot methods to detect the expression of GPX4 protein in activated HSC-T6 cells. The results are shown in Figure 4H,I. Western blotting showed lower levels of GPX4 protein in activated HSC-T6 cells treated with CUR@RCA than in cells treated with CUR or other micelles (Figure 4H,I). RA-modified micelles downregulated GPX4, suggesting that micelles could target activated HSC-T6 cells and reduce their expression and secretion of GPX4. These results at the protein level were corroborated at the mRNA level (Figure 4C). Our observation that CUR@RCA downregulated GPX4 more significantly than did CUR or other micelles suggested that co-delivery of CUR and RCA synergistically attenuates GPX4 expression in activated HSC-T6 cells, which is beneficial for inhibiting the activation of HSC-T6 and alleviating hepatic fibrosis.

Furthermore, the ROS scavenging efficiency of different micelles to activated HSC-T6 cells was investigated, and the results of cellular ROS were observed by fluorescence imaging using the oxidant-sensitive dye DCFH-DA. As depicted in Figure 4G, ROS levels were significantly elevated in activated HSC-T6 cells treated with micelles compared with those treated with free CUR. Preliminary speculation suggested that micelles may promote ferroptosis by increasing intracellular ROS levels, which can alleviate hepatic fibrosis by eliminating some activated HSCs. According to the above comprehensive analysis, CUR@RCA micelles exhibit robust activated-HSC-T6-targeting capability and ferroptosis-promoting effects.

### 3.9. In Vivo Synergistic Anti-Fibrotic Efficacy

**Figure 5:**
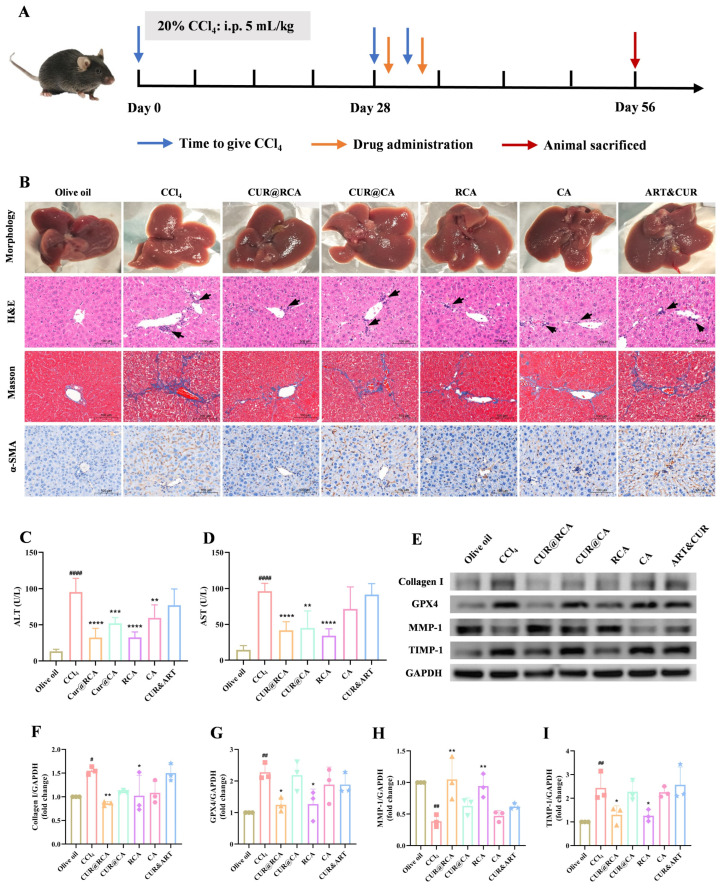
In vivo pharmacodynamic evaluation of micelles. (**A**) Schematic diagram of the drug administration protocol used in this study. (**B**) Gross morphology of liver and representative images of liver tissues stained with H&E, Masson, and immunohistochemical staining of α-SMA from different groups. Scale bars = 100 μm. (**C**) Serum ALT and (**D**) AST levels in mice following different drug treatments (n = 8). (**E**–**I**) The protein expressions of collagen I, GPX4, MMP-1 and TIMP-1 were assayed by Western blotting. GAPDH was included as loading control (n = 3). All data are shown as mean ± SD (compared with olive oil group, ^#^ *p* < 0.05; ^##^ *p* < 0.01; ^####^ *p* < 0.0001; compared with CCl_4_ group, * *p* < 0.05; ** *p* < 0.01; *** *p* < 0.001; **** *p* < 0.0001).

Based on the design of copolymer micelles and encouraged by the excellent CUR@RCA in vitro therapeutic results, the strong liver targeting capabilities, and the close correlation between ferroptosis and hepatic fibrosis, its therapeutic effects in CCl_4_-induced hepatic fibrosis mice were further evaluated. Figure 5A illustrates the process of establishing the hepatic fibrosis mice model and the treatment of micelles. Morphological observations revealed that the liver tissues of model mice exhibited an obvious granular appearance on the surface, thickened edges, and low elasticity, while CUR@RCA and RCA treatment significantly improved liver damage, as reflected by the liver becoming soft and smooth, without obvious granules (Figure 5B). Moreover, H&E staining further confirmed the recovery of liver injury. Following treatment with RCA and CUR@RCA, the inflammatory cell infiltration and necrosis of hepatocytes observed in the model group were significantly reduced, with almost no ectopic distribution noted in the healthy and CUR@RCA groups, indicating alleviation of hepatic fibrosis symptoms. Subsequently, Masson staining analyses were conducted to assess collagen deposition in the liver. As shown in Figure 5B, compared to healthy liver tissues, significant collagen deposition was noted following CCl_4_-induced hepatic fibrosis. In contrast, CUR@RCA, CUR@CA, RCA and CA were able to reduce the collagen fiber area to varying degrees. Notably, due to the surface targeting modification with VA and synergistic therapeutic advantages, CUR@RCA demonstrated the strongest efficacy in reducing collagen deposition in fibrotic livers, representing the optimal formulation for alleviating hepatic fibrosis. In Figure 5C,D, the levels of serum ALT and AST in mice receiving various treatments were suggested to vary. CUR@RCA and RCA effectively reduced the levels of ALT and AST in the serum, thereby restoring normal liver function.

As the activation of HSCs and collagen production are critical parameters that require elucidation during the progression of hepatic fibrosis, levels of α-SMA and collagen deposition in the livers of the different treatment groups were assessed. Immunohistochemical studies revealed that the CCl_4_-induced mice treated with CUR@RCA had decreased levels of α-SMA expression (Figure 5B). Additionally, Western blotting indicated that the levels of collagen I expression were also decreased in the CCl_4_-induced mice treated with CUR@RCA, suggesting that the process of hepatic fibrosis could be stopped by blocking the activation of HSCs (Figure 5E,F). Further, the release of MMP-1 in fibroblasts was enhanced by the treatment with CUR@RCA, while inhibiting the expression of TIMP-1, thereby promoting the degradation of ECM (Figure 5E,H,I). To further verify the regulatory effect of micelles on ferroptosis in the process of anti-hepatic fibrosis, levels of GPX4 in the livers of the different treatment groups were assessed. As shown in Figure 5E,G, the CCl_4_-induced mice treated with CUR@RCA had decreased levels of GPX4 expression, revealing that modulation of ferroptosis in HSCs contributed to improved hepatic fibrosis status.

Collectively, we evaluated hepatic fibrosis using a variety of methods, including immunohistochemical staining, Western blot measurement, and examination of serum ALT and AST levels to assess liver function. These findings indicate that CUR@RCA treatment can exert a positive anti-fibrotic effect by regulating ferroptosis, inhibiting HSC activation and restoring ECM homeostasis.

## 4. Conclusions

In this study, CA and RCA conjugate were synthesized by amido formation reaction for the oral delivery and hepatic fibrosis treatment of CUR. The FTIR and ^1^H-NMR spectra of CA and RCA indicated that ART and RA were grafted on COS, and the CUR-loaded micelles were prepared using the self-assembly method. The CUR@RCA micelles manifested small particle size, positive zeta potential, low CMC, and sustained in vitro release. In Caco-2 cells, the RA-mediated transportation and macropinocytosis/clathrin-mediated endocytosis route with energy consumption was revealed. In rats, the peak concentration (*C*_max_) and the oral relative bioavailability (*F_r_*) of CUR@RCA were 4.52-fold and 659.72% relative to the CUR crystals, respectively. These results demonstrate that chitosan oligosaccharide-based copolymer micelles functionalized with retinoic acid can serve as an effective delivery carrier for improving the solubility and oral bioavailability of hydrophobic molecules such as curcumin. In the in vitro efficacy evaluation, CUR-loaded micelles showed enhanced accumulation in activated HSC-T6 cells and regulated ferroptosis, which helped attenuate hepatic fibrosis. The mechanism of action is that CUR-loaded RCA inhibited the expression of GPX4, leading to excessive accumulation of intracellular ROS, ultimately regulating ferroptosis to achieve anti-fibrotic activity; specific rescue experiments are needed to further confirm the regulatory mechanism. Regarding in vivo synergistic anti-fibrotic efficacy, CUR-loaded RCA exhibited the ability to ameliorate hepatic fibrosis by degrading the fibrotic ECM and inhibiting HSC activation and proliferation. These findings will provide new therapeutic targets and inspiration for the treatment of hepatic fibrosis.

## Figures and Tables

**Table 1:** Oral pharmacokinetic parameters of CUR, CUR@CA, and CUR@RCA in rats.

Parameters	CUR	CUR@CA	CUR@RCA
*C*_max_ (ng/mL)	109.40	222.39	494.15
*T*_max_ (h)	2	4	2
*AUC*_(0−t)_ (ng/mL·h)	523.86	1563.14	3456.01
*MRT*_(0−t)_ (h)	4.21	7.36	6.31
*t*_1/2z_ (h)	2.80	6.01	2.95
*V*_z_/*F* (L/kg)	105.24	72.41	17.05
*CL*_z_/*F* (L/h/kg)	26.07	8.35	4.00
*Fr* (%)		298.39	659.72

## Data Availability

The original contributions presented in this study are included in the article. Further inquiries can be directed to the corresponding authors.

## Abbreviations

The following abbreviations are used in this manuscript:

EDCI	1-Ethyl-3-(3-dimethylaminopropyl)carbodiimide
DMAP	4-Dimethylaminopyridine
NHS	N-Hydroxysuccinimide
DMSO	Dimethyl Sulfoxide
α-SMA	α-Smooth Muscle Actin
Collagen I	Collagen Type I
GPX4	Glutathione Peroxidase 4
TIMP-1	Tissue Inhibitor of Metalloproteinases-1
MMP-1	Matrix Metalloproteinase-1
GAPDH	Glyceraldehyde-3-Phosphate Dehydrogenase
ROS	Reactive Oxygen Species
DAPI	4′,6-Diamidino-2-Phenylindole
RT–qPCR	Quantitative Reverse Transcription Polymerase Chain Reaction
Caco-2	Human Colon Adenocarcinoma Cell Line
HSC-T6	Hepatic Stellate Cell Line-T6
TGF-β1	Transforming Growth Factor-Beta 1
